# Public Policy and Constitutional Rights in Times of Crisis

**DOI:** 10.1017/S0008423920000256

**Published:** 2020-04-07

**Authors:** Emmett Macfarlane

**Affiliations:** University of Waterloo, Political Science, 200 University Ave. W., Waterloo, ON, N2L3G1

## Abstract

Federal and provincial policy responses to the COVID-19 pandemic raise a host of constitutional issues that decision makers must pay heed to or risk serious violations of individual rights under the Charter of Rights and Freedoms. This research note will examine a number of policy challenges as they relate to mobility rights (s. 6), legal rights (ss. 7 through 14), and equality rights (s. 15) and will articulate the factors that policy makers should consider in design and implementation. Other important constitutional questions, such as those relating to the division of powers, emergency powers and the relationship between the executive and Parliament, have also emerged in Canada but are beyond the scope of this note.

Federal and provincial policy responses to the COVID-19 pandemic raise a host of constitutional issues that decision makers must pay heed to or risk serious violations of individual rights under the Charter of Rights and Freedoms. This research note will examine a number of policy challenges as they relate to mobility rights (s. 6), legal rights (ss. 7 through 14), and equality rights (s. 15) and will articulate the factors that policy makers should consider in design and implementation. Other important constitutional questions, such as those relating to the division of powers, emergency powers and the relationship between the executive and Parliament, have also emerged in Canada but are beyond the scope of this note.

It is worth noting at the outset that from a constitutional law perspective, courts are likely to pay deference to legislative initiatives in the context of an emergency. Government policy objectives seeking to act in the interest of health and safety are rightly regarded as pressing and substantial, and any analysis of the reasonableness of limitations imposed on rights under section 1 of the Charter will begin with this consideration in mind. Yet, when coupled with the fact that the machinery of government is moving as quickly as possible to mitigate the impact of COVID-19 (both in terms of health and the economic fallout), this context makes it all the more important for policy makers to retain some vigilance regarding rights protection.

The most important factors in assessing the reasonableness of government policies will be to ensure restrictions are not arbitrary (that they are evidence based—or in the language of the judicial approach to section 1 under the “Oakes test,” that policies are rationally connected to their objectives) and that they are proportional in their effects. In what follows, I will briefly analyze a set of restrictive policies that have arisen in Canada in relation to particular rights and these two primary considerations. As the analysis will show, governments necessarily operate in a grey zone as they balance freedom with health and security concerns, but it is imperative that rights considerations are part of the (rapid) policy-making process currently underway. Attention to these factors will help to mitigate restrictions on the values we hold most dear.

## Borders and Travel Restrictions

Section 6 of the Charter guarantees every citizen the right to enter, remain in and leave Canada. Under s. 6(2), citizens also have the right to move to and take up residence in any province and “the right to pursue the gaining of a livelihood in any province.” Provincial power to regulate “reasonable residency requirements” for the receipt of social services, as well as any laws of general application (provided they do not discriminate primarily on the basis of residency), are protected under s. 6(3). Like all rights under the Charter, section 6 is subject to the reasonable limits provision of section 1. As one of the few Charter rights limited specifically to citizens, policy decisions such as turning away irregular migrants at the Canada-US border (Russell, 2020) do not have implications in the section 6 context (although that policy raises important human rights questions beyond the scope of this article).

There is relatively little caselaw under section 6, which may reflect the fact that it was never really conceived of as a broad freedom of movement provision (Green, 2014: 66–69). Courts have applied the provision to prevent the federal government from refusing to readmit citizens when there is no clear evidence that they pose a security risk (Federal Court, *Abdelrazik v. Canada*, 2009) but have upheld restrictions in other contexts, such that section 6 has not been interpreted to include the right to serve a prison sentence in Canada that was imposed in another country (Supreme Court of Canada, *Divito v. Canada (Public Safety and Emergency Preparedness)*, 2013).

There is scant evidence that the federal government has seriously considered refusing re-entry to citizens during the pandemic. Because it has the power to take much less rights-infringing measures, including imposing quarantine on returnees, such a drastic measure would certainly fail a reasonableness assessment.

A more challenging question concerns provincial authority to restrict entry to non-residents from other parts of Canada. Provinces like New Brunswick and Quebec recently announced that police would limit border crossings to essential traffic only (MacKinnon, 2020; Willing, 2020). Provinces have jurisdictional authority to act in the interest of health and safety, but these particular initiatives raise the question of discriminatory treatment on the basis of province of residency and thus may not be permissible under section 6(3) for that reason. In the midst of the crisis, governments have not done a particularly good job articulating the specifics of these policies, or their fundamental purpose. The Quebec policy, for example, appears to be an attempt to prevent the spread of the virus to regions of the province that lack the services to handle a significant surge in cases. Indeed, the government established intra-provincial checkpoints, not just checkpoints at the Quebec-Ontario border (Government of Quebec, 2020). To the extent that restricting movement applies both to residents within Quebec and to those attempting to come in from out of province, it is likely permissible under section 6.

As other commentators have noted, however, provinces that erect border crossings at inter-provincial highways may not be infringing the Charter but are likely acting *ultra vires* their authority under the constitutional division of powers (Dehaas, 2020). Only the federal government can directly regulate inter-provincial travel, although given the context around these initiatives, it is unlikely the provincial practices will be subject to constitutional challenge.

## Enforcing Quarantines and Regulating Gatherings

A set of legal rights under the Charter regulates police conduct and the administration of justice. Two of the most relevant provisions in the context of policies regulating personal conduct and movement are section 7's right to life, liberty and security of the person and section 9's right not to be arbitrarily detained or imprisoned. Section 15's equality rights guarantee might also be implicated in certain contexts.

The federal government has thus far refrained from asserting many of the broad powers it enjoys under the Emergencies Act or the Quarantine Act, but it is worth noting that the Charter context encouraged the creation of the Emergencies Act, which replaced the old War Measures Act. Two of the most fundamental changes are that under the Emergencies Act, a declaration of an emergency is reviewable by Parliament, and any temporary measures enacted under it are reviewable under the Charter. There is questionable benefit to use of the Emergencies Act during a pandemic, in part because so much of public health is governed at the provincial level. By contrast, the Quarantine Act has obvious relevance, and the constitutionality of some of its provisions—which would permit arrest for those refusing to self-isolate or permit the creation of quarantine zones, for example—remains unclear.

In the current crisis, returning travellers have, since March 25, been subject to a mandatory 14-day self-isolation period, under the Quarantine Act. The imposition of a broader domestic quarantine zone, such as to an entire city or affected region, would have to be applied under a strict assessment of evidence pertaining to geographic relevancy and the prevention of serious harm.

At the time of writing, there is little evidence that the health care system—or even specific hospitals—are overwhelmed, but given the experience in other countries, Canadian decision makers may have to account for this in the coming weeks. Nor have there been any major societal disruptions or widespread resistance to self-isolation messaging (although reports that some returning travellers have been going to grocery stores in violation of self-isolation guidelines is what precipitated the federal government's decision to invoke the Quarantine Act) (Connolly, 2020). Absent these factors, enforcing quarantines, along with the criminal penalties that might accompany them, would risk unreasonably violating sections 7 or 9 of the Charter. Section 7 permits the limitation of the right to life, liberty and security of the person and the right not to be deprived thereof “except in accordance with the principles of fundamental justice.” These principles include protections against laws that are overly broad in application, grossly disproportionate in the harms they impose relative to their benefits, or arbitrary. Limits on section 9 would face similar scrutiny under the Oakes test (particularly at the stage of assessing “minimal impairment” of the rights in question). The government has been prudent in refraining from exercising these powers too broadly.

On March 31, the province of Ontario announced new powers for police, requiring anyone facing charges under the province's emergency laws to identify themselves to police, under threat of serious fines (Freeman, 2020). The announcement was met on social media with alarm that the government was opening the door to widespread and unlimited carding—a practice also referred to as “street checks” and one that has been practised with well-established evidence of systemic racism (Tobias and Joseph, 2018). However, the policy as articulated appears only to apply to those being charged with an offence under the province's Emergency Management and Civil Protection Act (Government of Ontario, 2020). If the policy granted an unlimited power to require people to identify themselves to police, it would likely violate sections 7 and 9 of the Charter, as well as have obvious implications for equality rights. As a targeted and explicitly temporary grant of power, the new regulation is akin to existing law requiring that those charged with trespassing identify themselves, and it is likely a reasonable limit on legal rights.

## Economic Policy

Among the many initiatives announced thus far to mitigate the significant economic impact of the self-isolation measures has been the federal emergency wage subsidy (covering 75 per cent of salaries for all businesses that have lost at least 30 per cent of their revenue) and the Canada Emergency Response Benefit (a taxable benefit providing $2,000 per month for up to four months for workers who lose their income as a result of the pandemic). Provinces are developing their own policies. At the time of writing, there are several apparent gaps in these programs, including policies for students who may have lost summer jobs or imminent graduates about to enter the labour market.

These measures are unlikely to raise significant constitutional concerns, but policy makers should consider potential discriminatory effects in relation to eligibility criteria. Although the Charter has not generally been interpreted to protect economic or “positive” rights, once the government establishes a program, it must provide benefits in a non-discriminatory manner (Macfarlane, 2018). Among the protected grounds covered by section 15 is age. Governments are likely to enjoy significant latitude in carving out eligibility factors, particularly when considering career-stage factors that happen to correlate to age (Supreme Court of Canada, *Gosselin v. Quebec (Attorney General)*, 2002). Nonetheless, decision makers will want to evaluate eligibility criteria with some consideration of systemic impact along different dimensions, including gender or people with disabilities. Another important consideration is to ensure equitable treatment of Indigenous peoples, including those living in remote communities.

## Conclusion

In times of crisis or emergency, there are reasons to be concerned about government vigilance with respect to rights. Contexts like emergencies or security matters also may encourage courts to adopt a deferential or minimalist posture under the Charter (Macfarlane, 2012). Crises may even minimize litigation against constitutionally problematic policies. It is for this reason that Canadian policy makers need to be conscious of the Charter. While courts may be regarded as the “guardians” of the constitution, the executive and legislative branches enjoy a responsibility to uphold rights themselves (Hiebert, 2002; Baker, 2010). Even in the midst of a crisis and rapid deployment of new policies designed to mitigate the impacts of the COVID-19 pandemic, consideration of factors such as proportionality and ensuring policies are not arbitrary by holding fast to existing evidence can go some way to ensuring that limits on rights remain minimal and reasonable.
